# Comparative Genome Analysis of Three Thiocyanate Oxidizing *Thioalkalivibrio* Species Isolated from Soda Lakes

**DOI:** 10.3389/fmicb.2017.00254

**Published:** 2017-02-28

**Authors:** Tom Berben, Lex Overmars, Dimitry Y. Sorokin, Gerard Muyzer

**Affiliations:** ^1^Microbial Systems Ecology, Department of Aquatic Microbiology, Institute for Biodiversity and Ecosystem Dynamics, University of AmsterdamAmsterdam, Netherlands; ^2^Winogradsky Institute of Microbiology, Research Centre of Biotechnology, Russian Academy of SciencesMoscow, Russia; ^3^Department of Biotechnology, Delft University of TechnologyDelft, Netherlands

**Keywords:** chemolithoautotrophs, thiocyanate, *Thioalkalivibrio*, soda lakes, thiocyanate dehydrogenase

## Abstract

Thiocyanate is a C1 compound containing carbon, nitrogen, and sulfur. It is a (by)product in a number of natural and industrial processes. Because thiocyanate is toxic to many organisms, including humans, its removal from industrial waste streams is an important problem. Although a number of bacteria can use thiocyanate as a nitrogen source, only a few can use it as an electron donor. There are two distinct pathways to use thiocyanate: (i) the “carbonyl sulfide pathway,” which has been extensively studied, and (ii) the “cyanate pathway,” whose key enzyme, thiocyanate dehydrogenase, was recently purified and studied. Three species of *Thioalkalivibrio*, a group of haloalkaliphilic sulfur-oxidizing bacteria isolated from soda lakes, have been described as thiocyanate oxidizers: (i) *Thioalkalivibrio paradoxus* (“cyanate pathway”), (ii) *Thioalkalivibrio thiocyanoxidans* (“cyanate pathway”) and (iii) *Thioalkalivibrio thiocyanodenitrificans* (“carbonyl sulfide pathway”). In this study we provide a comparative genome analysis of these described thiocyanate oxidizers, with genomes ranging in size from 2.5 to 3.8 million base pairs. While focusing on thiocyanate degradation, we also analyzed the differences in sulfur, carbon, and nitrogen metabolism. We found that the thiocyanate dehydrogenase gene is present in 10 different *Thioalkalivibrio* strains, in two distinct genomic contexts/genotypes. The first genotype is defined by having genes for flavocytochrome *c* sulfide dehydrogenase upstream from the thiocyanate dehydrogenase operon (present in two strains including the type strain of *Tv. paradoxus*), whereas in the second genotype these genes are located downstream, together with two additional genes of unknown function (present in eight strains, including the type strains of *Tv. thiocyanoxidans*). Additionally, we found differences in the presence/absence of genes for various sulfur oxidation pathways, such as sulfide:quinone oxidoreductase, dissimilatory sulfite reductase, and sulfite dehydrogenase. One strain (*Tv. thiocyanodenitrificans*) lacks genes encoding a carbon concentrating mechanism and none of the investigated genomes were shown to contain known bicarbonate transporters. This study gives insight into the genomic variation of thiocyanate oxidizing bacteria and may lead to improvements in the application of these organisms in the bioremediation of industrial waste streams.

## Introduction

Thiocyanate is a pseudohalide C1 compound consisting of carbon, nitrogen, and sulfur. Natural sources include the process of cyanide detoxification, in which rhodanese (thiosulfate:cyanide sulfurtransferase, EC 2.8.1.1) catalyzes the transfer of the sulfane sulfur of thiosulfate to cyanide (Cipollone et al., 2007), and the breakdown of glucosinolate-containing plants, mostly members of the order *Brassicales* (Lee and Kwon, 2015). When cells of these plants are damaged, glucosinolates are hydrolyzed by myrosinase (thioglucosidase, EC 3.2.1.147) to form organic (iso)thiocyanates and organic nitriles, which are then further metabolized to form free thiocyanate (Lee and Kwon, 2015). In industry, it is found for example in gold and silver mine tailings, where cyanide is commonly used as a lixiviant, or in coking plant effluent (Gould et al., 2012). Thiocyanate is toxic to humans, inhibiting uptake of iodine by the thyroid gland (Brauer et al., 2006), and to numerous insects and aquatic organisms (Bhunia et al., 2000). Due to this toxicity, the removal of thiocyanate from industrial waste streams is an important process. Bioremediation has been suggested as an efficient and low-cost alternative to chemical destruction (Gould et al., 2012) and a number of applications have been devised and studied (Huddy et al., 2015; Kantor et al., 2015; reviewed in Watts and Moreau, 2016). This includes the coupling of lithotrophic thiocyanate degradation to algal lipid production (Ryu et al., 2014), transforming a waste stream into a potential product stream.

A number of bacterial species have been described with the ability to use thiocyanate for growth. Heterotrophic bacteria belonging to the genera *Klebsiella* (Lee et al., 2003), *Sphingomonas*, *Ralstonia* (du Plessis et al., 2001), *Methylobacterium* (Wood et al., 1998), and *Arthrobacter* (Betts et al., 1979) are capable of growth with thiocyanate as a nitrogen source. However, growth with thiocyanate as an electron donor is only possible for a small number of autotrophic sulfur-oxidizing bacteria: *Thiobacillus thioparus* (Katayama et al., 1998), *Tb. denitrificans* (Kelly and Wood, 2000), and *Paracoccus thiocyanatus* (Katayama et al., 1995) are all neutrophiles. Among extremophiles, there are the halophilic species *Thiohalophilus thiocyanoxidans* (Sorokin et al., 2007), and *Thiohalobacter thiocyanaticus* (Sorokin et al., 2010), *Halothiobacillus* sp. (Sorokin et al., 2014a) and the haloalkaliphilic species *Thioalkalivibrio paradoxus*, *Tv. thiocyanoxidans* (Sorokin et al., 2002) and *Tv. thiocyanodenitrificans* (Sorokin et al., 2004). There are two known enzymatic pathways for the primary degradation of thiocyanate (Sorokin et al., 2001b): the “COS pathway” and the “cyanate pathway.” The former involves hydrolysis of the C≡N bond by thiocyanate hydrolase (EC 3.5.5.8) to form carbonyl sulfide (COS) and ammonia. The COS is then further hydrolysed by COS hydrolase to sulfide, which enters the common sulfur oxidation pathway, and carbon dioxide. The latter pathway involves direct oxidation of the sulfane atom by TcDH (TcDH; thiocyanate:cytochrome *c* oxidoreductase), forming cyanate and elemental sulfur. The TcDH has recently been purified from *Tv. thiocyanooxidans* and *Tv. paradoxus* and fully characterized (Tsallagov et al., 2015).

The genomes of a large number of *Thioalkalivibrio* isolates, including the three known thiocyanate-utilizing species, were sequenced, assembled and annotated at the US Department of Energy by the Joint Genome Institute as part of its Community Science Program. It is the only genus of which multiple thiocyanate-oxidizing strains have been sequenced; the only other publicly available genome belongs to *Tb. thioparus* DSM 505, which uses the “COS pathway” (GenBank NZ_ARDU0000 0000.1).

*Thioalkalivibrio* is a group of chemolithoautotrophic, obligately haloalkaliphilic *Gammaproteobacteria* within the family *Ectothiorhodospiraceae*. *Thioalkalivibrio* are found in saline alkaline lakes occurring mostly in arid regions around the world, such as the Central Asian Great Steppes in southern Siberia, Mongolia and China, in the rain-shadowed areas of California and Nevada, Wadi Natrun in Egypt and the East African Rift Valley (Sorokin et al., 2014b). Soda lakes are characterized by a high pH (9–11) and moderate to high salinity, up to saturation. The dominant salts in solution are sodium carbonate/bicarbonate and NaCl, leading to a stable alkaline buffered system (Grant and Sorokin, 2011). Members of the genus *Thioalkalivibrio* are capable of oxidizing a variety of reduced, inorganic sulfur compounds, such as sulfide, polysulfides, thiosulfate, elemental sulfur, sulfite, and polythionates (Sorokin et al., 2001a). Three species can also use thiocyanate as an electron donor. *Tv. thiocyanoxidans* is a small motile vibrio and some strains (including type strain ARh 2^T^) are capable of growth in saturated soda brines (up to 4.3 M Na^+^) (Sorokin et al., 2002). The moderately salt-tolerant *Tv. paradoxus* is a large, non-motile rod and, in contrast to other members of the *Ectothiorhodospiraceae*, stores the elemental sulfur globules that are an intermediate of sulfide/thiosulfate/thiocyanate oxidation intracellularly (Sorokin et al., 2002). *Tv. thiocyanodenitrificans* is moderately salt-tolerant, the cells are motile rods and it is capable of anaerobic growth by denitrification with either thiosulfate or thiocyanate as electron donor (Sorokin et al., 2004). So far, genomic analysis of *Thioalkalivibrio* has been limited to *Tv. sulfidophilus* HL-EbGr7 and *Thioalkalivbrio* sp. K90mix (Muyzer et al., 2011a,b), two species not capable of metabolizing thiocyanate. Recently, the genome of *Tv. versutus* D301 was published (Mu et al., 2016), though it is unknown whether this strain is able to oxidize thiocyanate.

Here, we present a comprehensive comparative analysis of the genomes belonging to the type strains of thiocyanate-oxidizing *Thioalkalivibrio* species *Tv. paradoxus* ARh 1^T^, *Tv. thiocyanoxidans* ARh 2^T^ and *Tv. thiocyanodenitrificans* ARhD 1^T^. The main focus of the analysis was on thiocyanate degradation, as well as on core sulfur, carbon, and nitrogen metabolism, and genes involved in adaptation to haloalkaline environments. A comprehensive analysis of these genomes will add to our understanding of thiocyanate degradation both in natural systems, such as soda lakes, as well as in industrial applications.

## Materials and Methods

### Sequencing and Annotation

Sequencing and annotation procedures were described in detail elsewhere (Berben et al., 2015a,b,c). Basic genome properties are summarized in **Table 1**. *Tv. paradoxus* (GenBank accession CP007029.1) is a finished-quality genome consisting of 3,756,729 bp with a GC-content of 66.6%, whereas *Tv. thiocyanoxidans* (GenBank accession ARQK00000000.1) and *Tv. thiocyanodenitrificans* (GenBank accession AQZO00000000.1) are in permanent draft status. The genome for *Tv. thiocyanoxidans* contains 2,765,337 bp in 61 scaffolds, with a GC-content of 66.2%. The *Tv. thiocyanodenitrificans* genome consists of 3,746,647bp in three scaffolds, with a GC-content of 64.8%.

**Table 1:** Overview statistics for the genomes of *Tv. paradoxus, Tv. Thiocyanodenitrificans*, and *Tv. thiocyanoxidans*.

Species	Genome size (Mbp)	Scaffolds	% GC	Protein coding genes
*Tv. paradoxus* ARh 1^T^	3.8	1	66.6	3,557
*Tv. thiocyanodenitrificans* ARhD 1^T^	3.7	3	64.8	3,558
*Tv. thiocyanoxidans* ARh 2^T^	2.5	61	66.18	2,677

### General Analysis

Pairwise synteny was calculated for all pairs of genomes using the software SyMAP v4.2 (Soderlund et al., 2006) with default settings. Synteny blocks were exported from the program in tab-separated value format for further processing. BioPython 1.63 (Cock et al., 2009) (with Python 2.7.6) was used to calculate GC skew in all three genomes. Genomic islands in the *Tv. thiocyanodenitrificans* genome were identified by the program IslandViewer (Langille and Brinkman, 2009). Raw reads were aligned to the available assembly using Bowtie 2.1.0 (Langmead and Salzberg, 2012), the output was converted to BAM format, sorted and indexed using Samtools 0.1.19 (Li et al., 2009) and visualized in the Integrative Genomics Viewer 2.3.18 (Thorvaldsdóttir et al., 2013). Additionally, CheckM 0.9.7 (Parks et al., 2014) was used to assess the completeness and heterogeneity of the genomes. Visualization of the synteny, GC skew and genomic islands was accomplished using Circos 0.64 (Krzywinski et al., 2009).

### Detection of Specific Genes and Gene Clusters

The Basic Local Alignment Search Tool (BLAST) (Altschul et al., 1990) was used to align sequences of reference proteins to the genomes. Reviewed reference sequences were taken from UniProt^1^ database if available; otherwise non-curated sequences from TrEMBL were used instead. In case sequences from multiple organisms were available, the most closely related organism was chosen. The best hit in BLAST was used, if the query coverage exceeded 75% and the identity was higher than 30%. When a cluster of genes was expected, but no BLAST hits meeting the threshold were generated, the gene context of the cluster was inspected using the gene cassette search tool in IMG (Markowitz et al., 2014), which allowed for the detection of genes present, but below threshold. All reference sequences used, together with BLAST results for all strains studied here are available in the **Supplementary Table S1**.

### Phylogenetic Analyses

To determine the type of RuBisCO, sequences of the large subunit were taken from (Tabita et al., 2007) and aligned using Clustal Omega (Sievers et al., 2011). The optimal amino acid substitution model was determined using MEGA6 (Tamura et al., 2013) and phylogenetic trees were calculated with maximum likelihood. Statistical support for tree nodes was calculated with 500 bootstrap iterations. Sequences for the *dsr*AB tree were taken from Loy et al. (2009). This tree was constructed following the same procedure as described for RuBisCO. The trees were visualized using Figtree 1.4.2^2^.

## Results and Discussion

### General Genome Features

**FIGURE 1:**
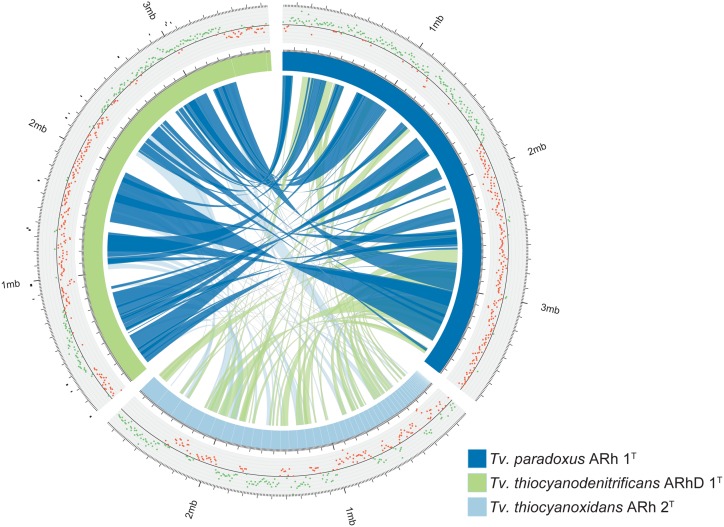
**Overview of the genomes of *Tv. paradoxus, Tv. thiocyanoxidans*, and *Tv. thiocyanodenitrificans*.** The blue and green segments represent the chromosomes, with white lines marking the contig boundaries. The inner ribbons connect pairwise synteny blocks, with wider ribbons denoting larger blocks. The plot outside the chromosome ring shows the GC-skew, the green and red dots denote positive and negative skew, respectively. The black markers outside the *Tv. thiocyanodenitrificans* chromosome indicate putative genomic islands, as predicted by IslandViewer.

The genome of *Tv. paradoxus* has been fully resolved and comprises approximately 3.7 Mbp. The genome of *Tv. thiocyanodenitrificans* consists of three scaffolds with a total size of 3.7 Mbp, of which the largest scaffold contains 90% of the sequence. The *Tv. thiocyanoxidans* sequence is divided over 61 contigs that remain unordered. Syntenic regions between *Tv. paradoxus* and *Tv. thiocyanodenitrificans* were apparent, with synteny blocks as large as 500 Kbp (**Figure 1**). The calculation of synteny between *Tv. thiocyanoxidans* and the other genomes is difficult, due to the limits imposed by contig sizes. Likely, this plot underrepresents the similarities between *Tv. thiocyanoxidans* and the other two genomes. Despite this, there are significant portions of sequence on all three genomes that are not conserved, including several complete contigs of *Tv. thiocyanoxidans*. The 300 Kbp scaffold of *Tv. thiocyanodenitrificans* has no synteny with either of the other genomes, whereas the second largest non-syntenic area is approximately 150 Kbp big. Upon closer inspection, this scaffold was revealed to contain 37 tRNA genes (comprising all 20 types) and two transposases; the rest of the gene products were annotated as hypothetical proteins. A complete set of tRNA genes is also present in the 3.4 Mbp scaffold. This scaffold has an average coverage of about 6,000x and few single nucleotide polymorphisms (SNPs), whereas the 300 Kbp scaffold has a coverage of 4,000x and shows a higher occurrence of SNPs. The origin of the two smaller scaffolds (300 and 30 Kbp, respectively) remains a question. The fact that they have no synteny with any of the other *Thioalkalivibrio* genomes indicates a possible contamination in the sequenced DNA. We performed a quality control step using CheckM (Parks et al., 2014), comparing the results for all three scaffolds combined vs. only the 3.4 Mbp scaffold. The results are summarized in **Table 2**. The CheckM analysis supports the reliability of scaffold one, as strain completeness (99.94%) does not decrease when scaffolds two and three are removed, whereas strain heterogeneity and contamination scores are significantly lower (0 vs. 40% and 1.03 vs. 2.26%, respectively). The strain completeness further shows that the main 3.4 Mbp scaffold has a (nearly) complete set of conserved marker genes. Given the information on the *Tv. thiocyanodenitrificans* sequence provided by the coverage and the CheckM analysis, only the largest scaffold (3.4 Mbp, scaffold 1) was used for further analysis.

**Table 2:** Overview of CheckM results.

Species	Scaffolds	Completeness	Contamination	Strain heterogeneity
*Tv. paradoxus* ARh 1^T^	All	99.97	0.02	0
*Tv. thiocyanodenitrificans* ARhD 1^T^	All	99.94	2.26	40
	Only scaffold 1	99.94	1.03	0
*Tv. thiocyanoxidans* ARh 2^T^	All	99.94	0.34	0

*CheckM (Parks et al., 2014) was used to estimate the completeness, strain heterogeneity and level of contamination. For Tv. thiocyanodenitrificans, there is an entry for the three scaffolds together as well as one for the largest scaffold (3.4 Mbp) alone.*

GC-skew provides an unambiguous estimate for the location of the origin of replication in *Tv. paradoxus*. The GC-skew of the *Tv. thiocyanodenitrificans* genome contains three sign changes. IslandViewer was used to predict genomic islands in this genome. The putative islands are small (**Figure 1**), but one is located in close proximity to the skew sign change at 250 Kbp. A transposase can indeed be found in the vicinity of this region. *dnaA* is located in the vicinity of the sign change at 2.3 Mbp. This suggests that the origin of replication should be located near this position.

### Thiocyanate Metabolism

**FIGURE 2:**
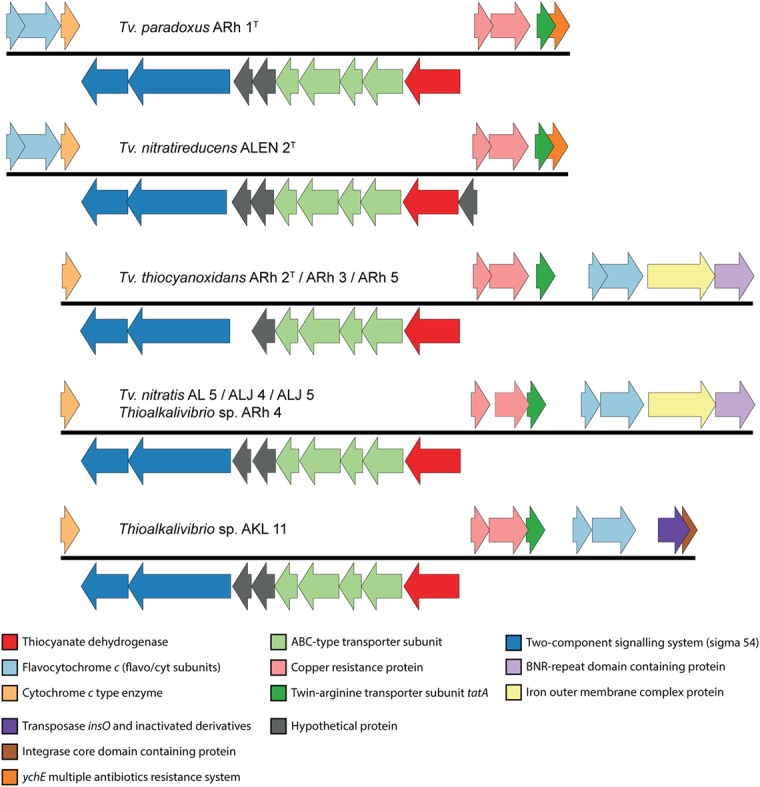
**Genomic context of the TcDH gene in all *Thioalkalivibrio* species in which it is present.** There are two main genotypes, defined primarily by the location of genes for flavocytochrome *c* sulfide dehydrogenase and the presence/absence of an iron outer membrane complex gene and a bacterial neuraminidase (BNR) gene. A third genotype is formed by the insertion of a transposase in *Thioalkalivibrio* sp. AKL 11. The IMG locus tags for the genes shown here are: ARh 1, Thith2703-2718; ALEN 2, TVNIR_2749-2765; ARh 2, G372DRAFT_01361-01376; ARh 3, C995DRAFT_1535-1550; ARh 5, G331DRAFT_0046-0061; AL 5, F574DRAFT_2579-2595; ALJ 4, C936DRAFT_2816-2832; ALJ 5, C937DRAFT_2833-2849; ARh 4, F465DRAFT_2655-2671; AKL 11, D574DRAFT_00079-00095.

The first description of thiocyanate dehydrogenase activity was provided by Sorokin et al. (2001b) who showed its presence and activity in the soluble fraction of lysed cells of *Tv. paradoxus* ARh 1 and *Thioalkalivibrio* sp. ARh 4. The degradation of thiocyanate by these bacteria correlated with the presence of a band at approximately 50 kDa after gel electrophoresis of total protein extracts (Sorokin et al., 2001b). The structure and sequence of TcDH, purified from *Tv. paradoxus* and *Tv. thiocyanoxidans*, were determined (Tsallagov et al., 2015) and this data has recently been released in the Protein Data Bank (PDB) under PDB IDs 5F75 (*Tv. paradoxus*) and 5F30 (*Tv. thiocyanoxidans*). The elucidation of the amino acid sequence of TcDH has enabled a search for the corresponding gene in all genomes that are currently available. TcDH is present in the two known cyanate pathway-utilizing *Thioalkalivibrio*, and its exact match is also present in eight genomes belonging to other *Thioalkalivibrio* strains not previously reported to be capable of thiocyanate oxidation. In contrast, no closely related genes can be found in any other publically available genomes in GenBank. The closest are two proteins with unknown function in the (meta)genomes of *Thioploca ingrica* and *Hydrogenobacter thermophilus*. The gene encoding TcDH is located in two different types of gene clusters, as shown in **Figure 2**. In *Tv. paradoxus* ARh 1^T^ and *Tv. nitratireducens* ALEN 2^T^ [which form a separate phylogenetic cluster within the genus *Thioalkalivibrio* (Tourova et al., 2007)] *TcDH* is followed downstream by four ABC-type transporter subunits (two permease and two ATP-binding), two hypothetical proteins and two genes annotated as a putative two-component regulatory system with a σ^54^ responsive element. In ALEN 2^T^, *TcDH* is flanked upstream by an additional gene of unknown function. On the opposite strand this cluster is flanked downstream by three genes coding for both subunits of flavocytochrome *c* sulfide dehydrogenase (*fcc*AB) and a small *c*-type cytochrome, and upstream by copper resistance genes *cop*CD, twin-arginine transporter system subunit *tat*A, and a gene annotated as multiple antibiotics resistance system *ych*E. The latter is actually more similar to the *Escherichia coli* gene *mar*C, whose function is currently unknown (McDermott et al., 2008). The second type of cluster is present in the second, major group of the TcDH-positive *Thioalkalivibrio*, including in *Tv. thiocyanoxidans* ARh 2^T^/3/4/5; *Tv. nitratis* ALJ4/5, AL5 and *Thioalkalivibrio* sp. AKL 11. Similar to the first, *TcDH* is followed downstream by four ABC transporter subunits, two genes of unknown function (one in the *Tv. thiocyanoxidans* strains) and a putative σ^54^ responsive two-component regulator. On the opposite strand this operon is flanked downstream only by a small *c*-type cytochrome and upstream by *cop*CD, *tat*A, *fcc*AB, a gene coding for an iron complex outer membrane receptor protein, and a bacterial neuraminidase (BNR) domain-containing protein. In *Thioalkalivibrio* sp. AKL 11, the latter two genes are missing. The flavocytochrome *c* genes are instead followed by genes encoding a transposase and an integrase core domain containing protein. Interestingly, strain AKL 11 was the most difficult of all TcDH-positive ones to adapt to growth on thiocyanate. These three groups of gene clustering also correspond to the three subgroups of protein homology of TcDH itself (Tsallagov et al., 2015).

TcDH is only active when it binds copper as a cofactor (Tsallagov et al., 2015), which might explain the presence of *cop*CD in the cluster. The twin-arginine transporter (tat) system transports folded proteins, including cofactors, across the cytoplasmic membrane. TcDH contains the C-terminal tat signal peptide [ST]-R-R-x-F-L-K, starting at amino acid position 34 (S-R-R-K-F-L-K), conserved for all 11 sequences known presently. The flavocytochrome *c* sulfide dehydrogenase participates in sulfide oxidation and the small *c*-type cytochrome possibly participates in the final stage of electron transport. The function of the ABC-type transporter might be in copper acquisition, while the role of the BNR-repeat containing gene and the iron outer membrane complex are currently unknown.

**FIGURE 3:**
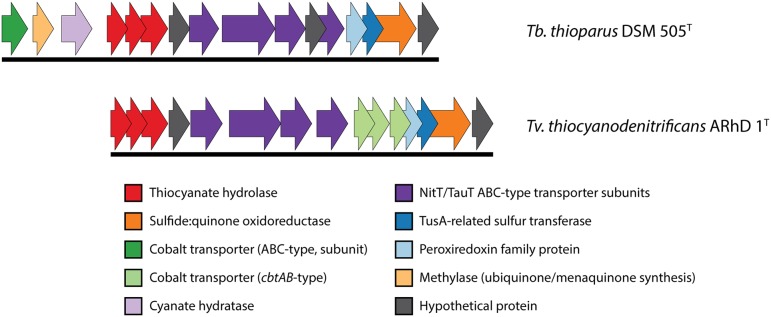
**Genomic context of thiocyanate hydrolase in *Thiobacillus thioparus* DSM 505 and *Tv. thiocyanodenitrificans*.** The genomic layout is similar in both species. The main differences that were found here are the different cobalt uptake systems and the presence of a cyanase gene directly preceding the thiocyanate hydrolase genes in *Tb. thioparus* DSM 505. The IMG locus tags for the genes shown here are: *Tb. thioparus*, B058DRAFT_00790-00805; *Tv. thiocyanodenitrificans*, ThithiDRAFT_0843-0857.

The genomic information on the COS pathway of thiocyanate oxidation is limited to two species: *Tb. thioparus* DSM 505^T^ and *Tv. thiocyanodenitrificans* ARhD 1^T^, as shown in **Figure 3**. In *Thiobacillus*, the three genes for thiocyanate hydrolase (*scn*ABC) are followed downstream by a hypothetical protein, four subunits of a NitT/TauT family ABC-type transporter (with another hypothetical protein between the third and fourth gene), a peroxiredoxin (a DsrE/F-like protein, often works in concert with TusA), a *tus*A-related sulfur transferase (which might be involved in elemental sulfur oxidation), sulfide:quinone oxidoreductase (which oxidizes sulfide), and finally another hypothetical protein. Genes for a cyanase, a methylase and a single ABC-type cobalt transporter subunit are found upstream of thiocyanate hydrolase. The presence of cyanase has also been found biochemically in another recently described SOB oxidizing thiocyanate via the COS pathway – *Thiohalophilus thiocyanoxidans* (Bezsudnova et al., 2007). This once again highlights that the early identification of the “cyanate pathway” on the basis of the presence of cyanase activity (Youatt, 1954) is not valid.

### Sulfur Metabolism

**FIGURE 4:**
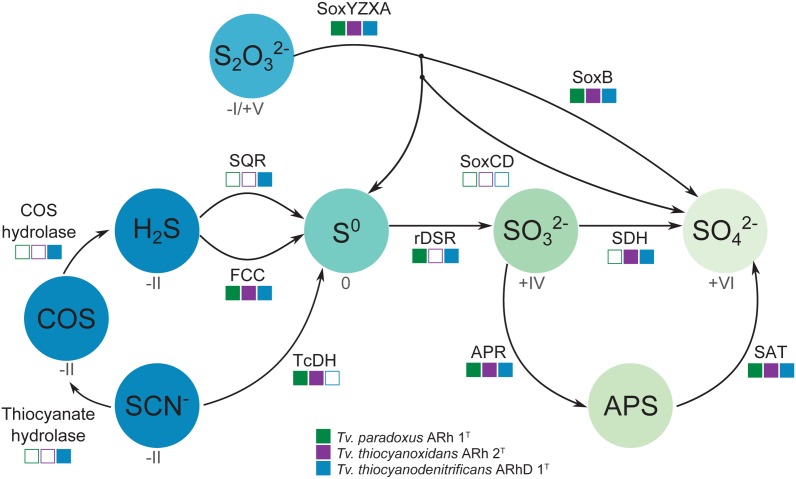
**Schematic overview of sulfur oxidation reactions in thiocyanate-oxidizing Thioalkalivibrio.** Thiocyanate is either degraded to hydrogen sulfide via the carbonyl sulfide pathway (thiocyanate hydrolase to COS, then COS hydrolase to H_2_S), or oxidized to the level of elemental sulfur via the cyanate pathway (TcDH; TCDH). Sulfide can be oxidized to elemental sulfur by flavocytochrome c (FCC) or sulfide:quinone reductase (SQR). Thiosulfate is covalently bound to SoxYZ by SoxXA, after which SoxB cleaves the sulfane sulfur to form sulfate; the sulfane sulfur can be oxidized to sulfate by SoxCD or released as elemental sulfur. Zero-valent sulfur is oxidized to sulfite by the reverse dissimilatory sulfite reductase system (DSR) after which the sulfite is finally oxidized to sulfate directly (SDH) or indirectly (Adenylyl Phosposulfate Reductase – APR – to APS, then Sulfate Adenylyltransferase – SAT – to sulfate). The color gradient represents the oxidation state for each component (if known), from fully reduced (–II, blue) to fully oxidized (+IV, light green). The squares indicate the presence (solid) or absence (hollow) of each enzyme system in the three *Thioalkalivibrio* species studied here.

The dissimilatory sulfur cycle is a complex network of red-ox reactions and intermediates, with many different enzymes involved in biological sulfur transformations. **Figure 4** shows a schematic overview of sulfur oxidation reactions in the *Thioalkalivibrio* species studied here, showing the presence or absence of enzymes per species.

**FIGURE 5:**
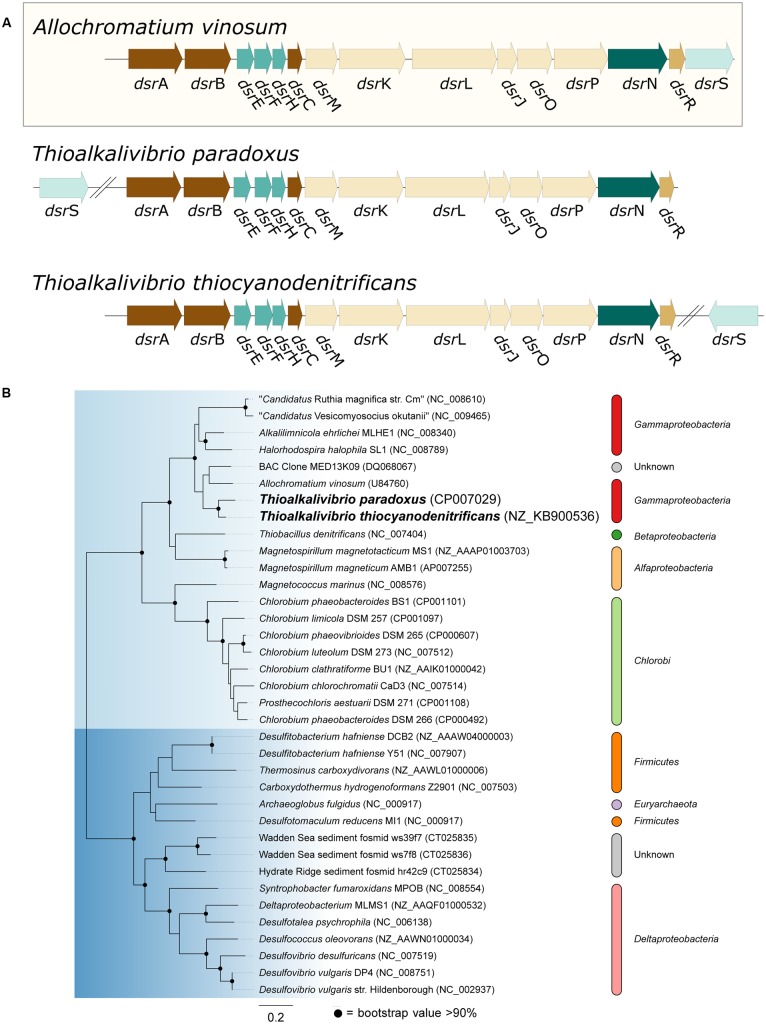
**Genes from the dissimilatory sulfite reductase pathway detected in the Thioalkalivibrio genomes. (A)** Comparison of the *dsr* cluster topology of *Tv. paradoxus* and *Tv. thiocyanodenitrificans* to that of the reference organism *Allochromatium vinosum*. **(B)** phylogenetic analysis of dsrAB sequences. The light blue clade comprises the dsrAB sequences from known sulfur-oxidizers, including *Tv. paradoxus* and *Tv. thiocyanodenitrificans*. The dark blue clade contains sequences from known sulfate-reducing bacteria. Sequences from *Pyrobaculum* and *Moorella* were used as the outgroup, but pruned from the tree.

Oxidation of sulfide is mediated by either flavocytochrome *c* (*fcc*AB) or sulfide:quinone reductase (*sqr*), both of which produce polysulfide. In all three *Thioalkalivibrio* genomes, *fcc*AB is detected, whereas *sqr* is detected only in *Tv. thiocyanodenitrificans* (52% identity at the amino acid level to *Acidithiobacillus ferrooxidans*) and with low sequence similarity in *Tv. paradoxus* (25% identity in amino acid sequence). In gammaproteobacterial sulfur-oxidizing chemolithotrophs, thiosulfate is usually oxidized to sulfur by a truncated Sox system, consisting of *sox*XAYZB, that lacks *sox*CD (Ghosh and Dam, 2009). This truncated system was also found in the genomes of all three thiocyanate-oxidizing *Thioalkalivibrio* species. From zero-valent sulfur, there are two pathways to sulfate, i.e., (i) a direct reaction in which *sox*CD performs a six-electron oxidation and (ii) a pathway where sulfur is oxidized to sulfite by the dissimilatory sulfite reductase pathway acting in reverse. The *dsr*ABEFHCMKLJOPNR cluster was found in *Tv. paradoxus* and *Tv. thiocyanodenitrificans*, with *dsr*S located elsewhere in the genome (**Figure 5A**). **Figure 5B** shows a phylogenetic analysis of concatenated *dsr*AB amino acid sequences. This analysis clearly shows that the *dsr* genes in these *Thioalkalivibrio* species belong to the clade of r*dsr*AB proteins with known sulfur-oxidizing activity (Loy et al., 2009). *Tv. thiocyanoxidans*, similar to *Thioalkalivibrio* sp. K90mix, lacks both the *dsr* cluster and *sox*CD (Muyzer et al., 2011b). From sulfite there is a direct pathway to sulfate via a two-electron transfer mediated by sulfite:cytochrome *c* oxidoreductase (Kappler and Dahl, 2001), which was only found in *Tv. thiocyanoxidans* and *Tv. thiocyanodenitrificans*, albeit with low similarity to the reference sequence from *Starkya novella* (35% identity in amino acid sequence). The alternative pathway uses adenylyl phosphosulfate (APS) as an intermediate and is catalyzed by adenylylsulfate reductase (*apr*AB) and sulfate adenylyltransferase (*sat*), both found in all three genomes.

Of the three thiocyanate-oxidizing type strains, *Tv. paradoxus* and *Tv. thiocyanodenitrificans* possess a set of genes that provides a complete metabolic pathway for the oxidation of reduced sulfur compounds to sulfate. The enzyme complex responsible for the conversion of [S^o^] to sulfite in *Tv. thiocyanoxidans* is presently unknown. It has been proposed that a system similar to cytoplasmic heterodisulfide reductase (*hdr*ABC), present in archaea and many anaerobic bacteria, functionally replaces *dsr*, and indeed *hdr*-like genes are found in many genomes that lack *dsr* including the genome of *Tv. thiocyanoxidans*, but evidence for its *in vivo* function is not available at present (Quatrini et al., 2009; Dahl, 2015).

### Carbon Metabolism

**FIGURE 6:**
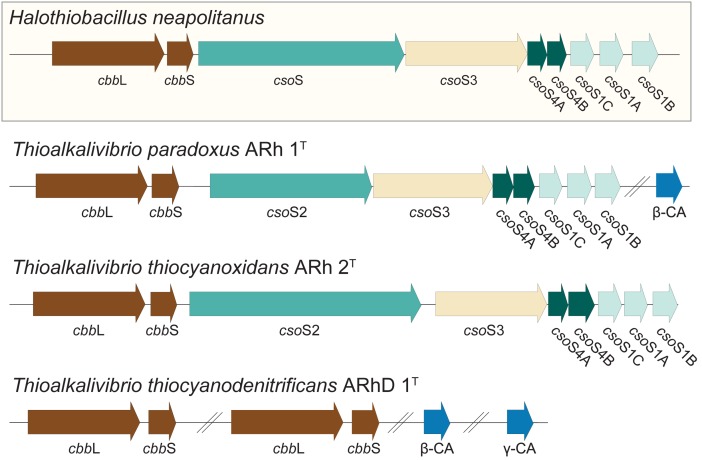
**RuBisCO and carboxysome genes detected in the *Thioalkalivibrio* genomes.** The genes for RuBisCO and carboxysome formation were compared to the genes from the model organism *Halothiobacillus neapolitanus*. *Cbb*L/S: respectively large and small subunits of RuBisCO; *Cso*S1A/B/C: major carboxysome shell proteins (due to high similarity these cannot be distinguished by BLAST, therefore the order in which they appear in *H. neapolitanus* is assumed); *Cso*S2: structural protein; *Cso*S3: carboxysome shell carbonic anhydrase; *Cso*S4A/B: structural proteins; CA: carbonic anhydrase not associated with carboxysomes. The IMG locus tags for the genes shown here are: *Tv. paradoxus*, Thith_2516-2524 (carboxysome cluster) and Thith_2150 (carbonic anhydrase); *Tv. thiocyanoxidans*, G372DRAFT_00040-0048; *Tv. thiocyanodenitrificans*, ThithiDRAFT_0610-0611 (*cbb*LS 1), ThithiDRAFT_2059-2060 (*cbb*LS 2), ThithiDRAFT_0715 (β-type carbonic anhydrase) and ThithiDRAFT_2393 (γ-type carbonic anhydrase).

It was previously shown, in a culture independent study of RuBisCO diversity in soda lakes, that *Thioalkalivibrio* contain RuBisCO of type Ia (Kovaleva et al., 2011). Many lithoautotrophic bacteria possess the capability to produce carboxysomes, specialized bacterial micro-compartments containing a high concentration of RuBisCO and carbonic anhydrase, the enzyme interconverting CO_2_ and HCO_3_^−^ (Heinhorst et al., 2014). Correspondingly, a search for these genes in *Thioalkalivibrio* was performed using sequences from the model gammaproteobacterial SOB *Halothiobacillus neapolitanus* (Cannon et al., 2003) as a reference; the results are shown in **Figure 6**. Both *Tv. paradoxus* and *Tv. thiocyanoxidans* possess RuBisCO small and large subunits (*cbb*S*, cbb*L*)*, and a complete cluster of alpha carboxysome genes: carbonic anhydrase *cso*S3 and carboxysome shell proteins *cso*S2, *cso*S4A/B and *cso*S1A/B/C, with high similarity to those found in *H. neapolitanus*. *Tv. paradoxus* has an additional beta-type carbonic anhydrase gene. The *Tv. thiocyanodenitrificans* genome contains only the genes for RuBisCO (two copies of each subunit) and two non-carboxysome-associated carbonic anhydrases, one beta- and one gamma-class. However, it lacks the genes that are involved in the formation of the carboxysomes, which is consistent with previous electron microscopy data indicating the absence of carboxysomes in this bacterium (Sorokin et al., 2013). This is an interesting observation, as it implies that *Tv. thiocyanodenitrificans* has a strong disadvantage in the competition for inorganic carbon. Previous studies in cyanobacteria have shown that the lack of a functional carboxysome leads to phenotypes that require significantly higher concentrations of CO_2_ (Price and Badger, 1989; Rae et al., 2013). Despite the fact that soda lakes have a large concentration of inorganic carbon (though, depending on the pH, this may exist primarily as unavailable CO_3_^2−^), the fact that other *Thioalkalivibrio* do form carboxysomes (Sorokin et al., 2013) would suggest that *Tv. thiocyanodenitrificans* must have a strategy to prevent it from being out-competed. Its ability to grow anaerobically under denitrifying conditions, using thiosulfate or thiocyanate as electron donor might provide a niche where it can escape competition.

Specific transport enzymes for bicarbonate, such as SbtA and BicA known from alkalitolerant and haloalkaliphilic cyanobacteria (Sandrini et al., 2014; Kupriyanova and Samylina, 2015), were not detected in the investigated *Thioalkalivibrio* species. For SbtA it was previously shown that at least one other *Thioalkalivibrio* species, *Tv. sulfidophilus*, does not possess this gene, whereas *Thioalkalivibrio* sp. K90mix does (Muyzer et al., 2011a,b). A third bicarbonate uptake system from cyanobacteria, BCT1, is an ABC-type transporter. Although ABC-type transporters are detected in *Thioalkalivibrio*, it is not possible to infer their substrate from sequence information alone, which leaves bicarbonate transport in these organisms currently without a definitive explanation of the mechanism. A role for extracellular carbonic anhydrases in the C_i_ acquisition at high pH was previously shown in the alkaliphilic cyanobacterium *Microcoleus chthonoplastes* (Kupriyanova et al., 2011). However, analysis of the amino acid sequences of the carbonic anhydrases present in the genomes of *Tv. paradoxus* and *Tv. thiocyanodenitrificans* using SignalP (Petersen et al., 2011) and TMHMM (Krogh et al., 2001) revealed no protein export signal peptides or transmembrane helices.

### Nitrogen Metabolism

Complete denitrification is a multistep pathway from nitrate, via nitrite, nitric oxide and nitrous oxide, to molecular nitrogen (N_2_) (Zumft, 1997). *Tv. thiocyanodenitrificans* is unique among the whole *Thioalkalivibrio* genus, being capable of complete denitrification (Sorokin et al., 2004). While some strains of *Tv. thiocyanoxidans* are capable of assimilatory nitrate reduction, neither *Tv. thiocyanoxidans* nor *Tv. paradoxus* is capable of using any of the NO_x_ as electron acceptor (Sorokin et al., 2002). Nevertheless, in all three genomes an operon encoding the membrane-bound dissimilatory nitrate reductase (*nar*GHIJ) is present (**Table 3**). In contrast, a putative cluster consisting of genes related to cytochrome *cd_1_*-containing nitrite reductase (*nir*S), was only detected in *Tv. thiocyanodenitrificans*, in accordance with the spectroscopic evidence of *nir*S presence in this species that was previously published (Sorokin et al., 2004). Interestingly, a putative nitric oxide reductase cluster, consisting of *nor*CBQD was detected in both *Tv. thiocyanodenitrificans* and *Tv. paradoxus* (**Table 3**). The same holds true for nitrous oxide reductase (**Table 3**). In *Tv. paradoxus* this cluster has a different arrangement compared to that of *Tv. thiocyanodenitrificans*, and it is located directly adjacent to the nitric oxide reductase cluster, rather than elsewhere in the genome. While the presence of a set of genes necessary for complete denitrification is consistent with the *Tv. thiocyanodenitrificans* phenotype, the *nar*, *nor* and *nos* gene clusters in *Tv. paradoxus* are apparently non-functional, since this organism is incapable of using NO_x_, both in assimilatory and dissimilatory modes (Sorokin et al., 2002).

**Table 3:** Presence of genes for denitrification in *Thioalkalivibrio*.

Species	Nitrate reductase (*nar*)	Nitrite reductase (*nir*)	Nitric oxide reductase (*nor*)	Nitrous oxide reductase (*nos*)
*Tv. paradoxus* ARh 1^T^	Yes	Partial	Yes	Yes
*Tv. thiocyanodenitrificans* ARhD 1^T^	Yes^∗^	Yes	Yes	Yes
*Tv. thiocyanoxidans* ARh 2^T^	Partial	Not found	Not found	Not found

*Genes were detected using protein BLAST using annotated reference genes from well-studied Gammaproteobacteria (see **Supplementary Table S1**), using coverage > 75% and identity > 30% as thresholds. ^∗^The presence of narI and narJ was confirmed by chromosomal cassette search in IMG as the BLAST search with Escherichia coli reference sequences did not find these genes.*

Since *Tv. paradoxus* is most closely related to *Tv. nitratireducens* (Sorokin et al., 2013), a member of a denitrifying consortium, which grows anaerobically by nitrate reduction to nitrite, but is incapable of further denitrification (Sorokin et al., 2003), it might be speculated that, perhaps, both species descended from a complete denitrifying ancestor but then evolved in different habitats.

### Adaptations to Haloalkaline Environments

Most salt-tolerant bacteria are capable of synthesizing organic compatible solutes to resist the osmotic pressure from the hypersaline environment. Previous studies on an extremely salt-tolerant *Thioalkalivibrio* sp. ALJ15, have shown that glycine betaine is the dominant solute produced, with sucrose as a minor fraction (Banciu et al., 2005). In all three thiocyanate-utilizing species, the BCCT type betaine transporter *bet*L (Ziegler et al., 2010) was detected, as well as genes for the synthesis of glycine betaine via sarcosine (glycine/sarcosine N-methyltransferase and sarcosine/dimethylglycine N-methyltransferase) (Nyyssölä et al., 2000). The key enzyme for glycine betaine production from choline, choline dehydrogenase, was not found in these species. Likewise, sucrose phosphate synthase was found in all three species. Key genes for ectoine biosynthesis, *ect*ABC, were not detected. These results are compatible with previous studies on the genomes of other *Thioalkalivibrio* type strains (Muyzer et al., 2011a,b).

In addition to the osmotic pressure, soda lakes are energetically challenging by imposition of an unfavorable pH gradient across the cell membrane. Possible adaptations to this include the use of sodium energetics rather than proton energetics, secondary sodium pumps, such as sodium-dependent sym- and antiporters, and structural adaptations, such as the incorporation of squalene and cardiolipin in the cell membrane to decrease the rate of proton leakage (Banciu and Muntyan, 2015). Genes for the primary sodium-pumping NADH:quinone oxidoreductase Na^+^-NQR, extensively studied in alkalitolerant *Vibrio* species (Barquera, 2014), were not detected in any of the three genomes. Instead, a complete cluster of genes for the ferredoxin:NAD^+^ oxidoreductase Rnf (*rnf*ABCDGE), of which a sodium-translocating variant was first described in *Acetobacterium woodii* (Biegel et al., 2011), was found in *Tv. paradoxus* and *Tv. thiocyanodenitrificans*. *Tv. thiocyanoxidans* was shown to only have *rnf*ABCE, lacking *rnf*DG. Unfortunately, to date, no data on conserved amino acid residues involved in sodium coordination has been published. This makes it impossible to infer the type of *rnf* (Na^+^ or H^+^ translocating) from the sequence alone. Recently, a first example of a sodium-pumping cytochrome *c* oxidase, type *cbb*_3_, from *Tv. versutus* AL 2^T^ was discovered (Muntyan et al., 2015). Detailed analysis of the protein sequence in that study revealed a number of amino acid residues that are essential to the sodium-pumping function of this enzyme. In the same study, it was shown that *Tv. paradoxus* ARh 1^T^ also possesses the Na^+^-pumping cytochrome *cbb*_3_, based on sequence information. A BLAST search of the three genomes in this study reveals that all three possess the Na^+^-translocating *cbb*_3_ cytochrome oxidase and that both *Tv. paradoxus* and *Tv. thiocyanodenitrificans* have an additional H^+^-translocating copy of this gene (Supplementary Figure S1). It was previously shown that *Thioalkalivibrio* do not possess sodium-driven ATP synthase genes (Hicks et al., 2010). A primary sodium cycle is still possible, with cytochrome *cbb*_3_ oxidase exporting sodium ions and a sodium driven *rnf* complex using the sodium motive force to drive the reverse electron flow for the NADH synthesis common in chemolithoautotrophic bacteria. However, at present, there is no experimental evidence for such a system.

Squalene, a neutral lipid, is an isoprenoid hydrocarbon that is a component in the cell membrane of some of the alkaliphilic bacteria in which it reduces proton leakage (Hauß et al., 2002). *Tv. paradoxus*, *Tv. thiocyanodenitrificans*, and *Tv. thiocyanoxidans* all possess a full set of genes (*dxs*, *dxr*, *isp*ACDEFGH) for producing squalene from glyceraldehyde-3-phosphate and pyruvate via the non-mevalonate pathway (Eisenreich et al., 2004). According to the total lipid analysis of *Tv. paradoxus*, lanosterol (a product of linear squalene cyclization) comprised approximately 50% of the total membrane lipids (our unpublished data). Cardiolipin is a negatively charged polar membrane phospholipid that can act as a proton trap, stabilizes respiratory complexes and is involved in the salt-stress response (Haines and Dencher, 2002; De Leo et al., 2009; Arias-Cartin et al., 2012), although recent research has shown that it is not absolutely required for oxidative phosphorylation in alkaliphilic *Bacillus pseudofirmus* (Liu et al., 2014). In bacteria it is synthesized from two phosphatidylglycerol molecules by cardiolipin synthase, an enzyme with two phospholipase D domains (Tian et al., 2012). Again, in all three species genes aligning to *Escherichia coli* cardiolipin synthase were found, containing two phospholipase D domains, albeit with low sequence similarity (25–38%). However, only *Tv. thiocyanoxidans* possesses three genes that distinctly align to the three subunits of cardiolipin synthase. In the other two species, only two genes were found.

## Conclusion

In this paper we describe the first comparative genomics study of three thiocyanate-oxidizing *Thioalkalivibrio* isolated from soda lakes. We show the presence of the recently described thiocyanate dehydrogenase in *Tv. thiocyanoxidans* and *Tv. paradoxus*, as well as in eight additional *Thioalkalivibrio* genomes. Analysis of the genomic context of this gene reveals the presence of genes for the uptake of a copper co-factor (*cop*CD) and for the transport of the enzyme to the periplasm (*tat*A), sulfide dehydrogenase (*fcc*AB) and a number of genes of unknown function. There are two genotypes of this cluster, whose distribution correlates with *Thioalkalivibrio* phylogenetic relatedness. Two genes are absent from this cluster in *Thioalkalivibrio* sp. AKL 11 and appear to decrease its ability to adapt to growth on thiocyanate, suggesting that this information could be used in for example strain selection for biotechnology applications. *Tv. thiocyanodenitrificans* utilizes the COS pathway and its genome correspondingly contains genes for thiocyanate hydrolase. *Tv. thiocyanoxidans* stands out from the other two for its lack of the rDSR pathway and the presence of a putative sulfite dehydrogenase. All three species fix carbon using type Ia RuBisCO and *Tv, thiocyanoxidans* and *Tv. paradoxus* additionally have genes for a carbon-concentrating mechanism. The absence of these genes in *Tv. thiocyanodenitrificans* suggests it uses a different competitive strategy.

## Author Contributions

TB performed the various analyses and drafted the manuscript, LO and GM assisted in the interpretation of results and, together with DS, provided critical review of the manuscript. All authors have read and approved the final version of the manuscript.

## Conflict of Interest Statement

The authors declare that the research was conducted in the absence of any commercial or financial relationships that could be construed as a potential conflict of interest.

## Footnotes

1 www.uniprot.org

2 http://tree.bio.ed.ac.uk/software/figtree/

## Supplementary Material

TABLE S1 **The list of reference sequences used for detecting genes in the three *Thioalkalivibrio* genomes and the associated BLAST results**.
