# Supplementary material for: Comparative Genome Analysis of Three Thiocyanate Oxidizing Thioalkalivibrio Species Isolated from Soda Lakes
Source: Front Microbiol. 2017 Feb 28;8:254. doi: 10.3389/fmicb.2017.00254 (PMC5328954; doi:10.3389/fmicb.2017.00254)
Supplement: Supplementary file 2 [file Image_1.pdf]

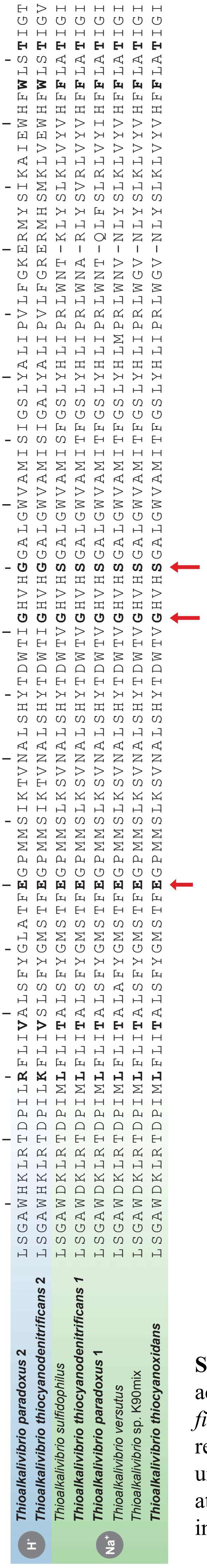

Supplementary Figure S1: Alignment of *Thioalkalivibrio* cytochrome *cbb*$_3$ oxidase amino acid sequences. The top two sequences, belonging to *Tv. paradoxus* and *Tv. thiocyanodenitrificans*, translocate protons. All the others translocate sodium ions. Residues printed in bold represent those described by Muntyan et al. (Muntyan et al., 2015), as conserved in sodium-translocating *cbb*$_3$ proteins (225L, 229T, 340E, 361G, 365S and 406T), except for those at position 406 – these show the W conserved in H+-translocating variants. The red arrows indicate residues forming the sodium channel.
